# Association of Vitamin D Level With Disease Severity and Quality of Life in Newly Diagnosed Patients of Ulcerative Colitis: A Cross-Sectional Analysis

**DOI:** 10.7759/cureus.16481

**Published:** 2021-07-19

**Authors:** Kaibalya R Dash, Chittaranjan Panda, Haribhakti S Das, Debakanta Mishra, Sambit Kumar Behera, Prashant K Parida, Debjyoti Mohapatra

**Affiliations:** 1 Gastroenterology, Srirama Chandra Bhanja Medical College and Hospital, Cuttack, IND; 2 Gastroenterology, Institute of Medical Sciences and SUM Hospital, Bhubaneswar, IND; 3 Community Medicine, Srirama Chandra Bhanja Medical College and Hospital, Cuttack, IND

**Keywords:** colitis, inflammation, gastro-intestinal, crp, bmi

## Abstract

Introduction

Ulcerative colitis is an immunologically mediated disorder affecting the gastrointestinal tract. Vitamin D3 has been shown to modulate many immunological diseases, but its role in ulcerative colitis is not well documented. This study was done to find out if levels of vitamin D are associated with the severity of disease and quality of life in ulcerative colitis patients.

Methods

This cross-sectional study consists of two parts. The first part consists of having a comparative assessment of baseline parameters of newly diagnosed ulcerative colitis patients and healthy controls. The 2nd part consists of an evaluation of the association of levels of vitamin D3 with disease severity and quality of life in ulcerative colitis. Independent predictors of disease severity and quality of life were assessed using multiple linear regression.

Results

Vitamin D levels were significantly lower in healthy controls compared to newly diagnosed ulcerative colitis patients. Median ulcerative colitis disease activity index score was significantly higher in the vitamin D deficient group compared with those who had normal vitamin D levels (p-0.001). Quality of life was also poor in the vitamin D deficient group compared to those with normal vitamin D levels (p-0.000). Vitamin D levels were found to be independent predictors of ulcerative colitis disease activity scores and health-related quality of life scores.

Conclusion

Vitamin D may have some immunomodulating properties, which might be associated with decreased ulcerative colitis disease activity index and better quality of life.

## Introduction

Ulcerative colitis and Crohn’s disease are chronic, relapsing, immunologically mediated disorders that are described together as inflammatory bowel diseases (IBD). Ulcerative colitis is a heterogeneous disease characterized by various genetic and environmental abnormalities that may cause T cells to mount an immune response against commensal intestinal bacteria and, in this process, may damage the intestinal lining. Studies have shown that the sequence of pathogenesis in ulcerative colitis starts with a genetic abnormality that causes an immune regulatory defect which may then be modified by an environmental trigger like a food-borne infection. Ulcerative colitis (UC) causes persistent mucosal inflammation of the colon, usually without granulomas on histology [1]. It rarely causes transmural involvement of the colon except in certain specific conditions like toxic megacolon. It affects the rectum and variable length of the colon continuously and is characterized by a relapsing and remitting course of disease. Dysregulated immune responses have recently been proven to have a definitive role in the development of ulcerative colitis [2]. A dysregulated innate and adaptive immunity causes increased production and release of cytokines, including both regulatory and inflammatory cytokines like TNF alpha, IL-2, IL-6, and IFN-α. These proinflammatory cytokines partake in maintaining the inflammatory response and lead to the uncontrolled intestinal inflammation seen in IBD. Vitamin D, especially its metabolite 1.25-dihydroxyvitamin D [1.25(OH)2D], is an important regulator of the immune system, and its deficiency has been proven to be related to altered immunity [3,4]. These immunological abnormalities are directly linked with the initiation and propagation of intestinal inflammation. The dendritic cell maturation is affected by vitamin D deficiency and may lead to decreased production of cytokine IL-12, a cytokine that is exclusively produced by dendritic cells. At the same time, it stimulates the release of inhibitory cytokine IL-10 and thereby modulates Tb cell function [3,4]. Vitamin D3 helps in the preferential differentiation of T cells into T regulatory cells [5]. Therefore the optimum level of vitamin D provides the first line of defense against pathogenic organisms and at the same time also prevents uncontrolled activation of the immune system, which may be detrimental. It has also been hypothesized that vitamin D affects the gut microbiome [6]. A large prospective study recently had shown that people with very low vitamin D levels were at risk of developing Crohn’s disease [7]. Even though the link between vitamin D and ulcerative colitis is not as strong as with Crohn’s, there are still a few studies that show an inverse relationship between vitamin D levels and disease severity [4,7]. A vitamin D serum level above a certain threshold limit is assumed to be critical to maintain a balanced immune response and to protect against intruding microorganisms without triggering auto-inflammation [6]. Vitamin D has a definite role in maintaining the integrity of the intestinal wall and preventing gut leakage [4]. Despite the well-recognized association between vitamin D deficiency and bone health in IBD, few studies have examined the immunomodulatory function of vitamin D by exploring its association with disease activity and health-related quality of life (HRQOL) in an IBD cohort, especially UC patients [8]. There are also limited clinical data on the effect of vitamin D replacement therapy on disease activity or health-related quality of life [8]. This study was primarily aimed to make a comparative assessment of the ulcerative colitis disease activity index and quality of life in newly diagnosed patients of ulcerative colitis with normal and deficient levels of serum vitamin D and to know whether vitamin D levels were independent predictors of disease activity and quality of life in such patients.

## Materials and methods

This cross-sectional study was conducted in the Department of Gastroenterology of SCB Medical College, a leading tertiary care teaching hospital in eastern India. This study was conducted for a period of 1 year, from January 2018 to December 2018.

This study was composed of two parts. The first part was a case-control study to compare various biochemical and inflammatory biomarker parameters among the ulcerative colitis cases and the controls without the disease. The second part comprised mainly the descriptive analysis of ulcerative colitis cases and assessment of ulcerative colitis disease activity index (UCDAI) and HRQOL and its association with vitamin D level of patients. UCDAI scoring is based on stool frequency, rectal bleeding, mucosal appearance during colonoscopy, and physician rating of the disease activity. The quality of life was assessed by a short inflammatory bowel disease questionnaire [9].

Selection of cases

Inclusion Criteria

Consecutive newly diagnosed ulcerative colitis patients who attended the outpatient department during the study time period and were aged more than 18 years were included in the study. The diagnosis of ulcerative colitis was done by the presence of the following criteria. A history of diarrhea or blood in stools for more than 4 weeks; macroscopic appearance by colonoscopy (granular mucosa with mucosal erythema and erosions, diffuse ulcerations with or without mucopurulent exudates, loss of light reflex and vascular pattern); and histological features on biopsy specimens compatible with ulcerative colitis.

Exclusion Criteria

The following criteria of patients were excluded from this study: the patient did not provide informed consent; patients with serious life-threatening, co-morbidities (as decided by treating physician), malignancy, renal or hepatic failure; patients who have been already supplemented with vitamin D within the past six months; pregnant and breastfeeding women. We also excluded patients with toxic megacolon and those presenting with acute severe colitis - these cases are generally referred to surgical gastroenterology immediately and therefore were excluded.

Selection of controls

Age (within 5 years of age of case) and sex-matched controls were selected from the outpatient department of gastroenterology. The selected controls had come for complaints that were not related to Inflammatory bowel disease, and they had no evidence of disease in the past. The exclusion criteria for controls were the same as the cases.

Assessment of cases and controls

All the cases and controls underwent detailed physical examination and laboratory investigation, which included inflammatory parameters like erythrocyte sedimentation rate and C reactive protein, liver function test, renal function test, serum vitamin D3 level, and inflammatory markers like C reactive protein, erythrocyte sedimentation rate. Colonoscopy was performed in all the cases to know the extent of the disease, but it was not done in the controls. Disease activity scores of the patients were calculated using the UCDAI. HRQOL was measured using the short inflammatory bowel disease questionnaire (SIBDQ) [9]. The SIBDQ is a simple 10-point questionnaire that has been used in previous studies and is a validated measure of HRQOL in ulcerative colitis patients. Vitamin D level assay was done using chemiluminescence immunoassay by the agappe reagents in COBAS e411 machine (Roche Diagnostics, Basel, Switzerland). We had taken a cut off of 30ng/dl for vitamin D deficiency [10].

Statistical analysis

The collected data were entered into an excel spreadsheet and analyzed using Statistical Package for the Social Sciences (SPSS) version 22.0 (IBM Inc., Armonk, New York). Descriptive data were expressed as means and standard deviation or median and interquartile range. Normality of data was assesses by Kolmogorov Smirnoff test. Unpaired t-test was used to compare the descriptive statistics of ulcerative colitis patients and control. The same test was used to compare the parameters of ulcerative colitis patients with normal vitamin D levels with those of vitamin D deficient patients. Median of disease activity scores and quality of life scores in the two groups of ulcerative colitis patients were compared using the Mann Whitney test. Categorical data were compared using the Chi-square test of association. Multiple linear regression analysis was done to find the independent predictors of disease activity and quality of life in ulcerative colitis patients.

Ethical Approval

The study was approved by the Institutional Ethics Committee. Those fulfilling the inclusion criteria were recruited after written informed consent. The study was conducted in full compliance with the guidelines of good clinical practice of the World Medical Assembly Declaration of Helsinki [11] and the Indian Council of Medical Research guidelines [12].

## Results

A total of 84 patients were diagnosed with ulcerative colitis during the study time period. Out of these, 76 fulfilled inclusion criteria and were enrolled in this study. 58 (76.3%) of them were male, and 16 (23.7%) were female. We had enrolled 76 age and sex-matched controls in this study. Baseline comparisons of cases and controls are illustrated in Table 1. Vitamin D levels in controls (39.89 ng/dl) were significantly high (p<0.03) compared to newly diagnosed ulcerative colitis patients (23.40 ng/dl). Other inflammatory markers were also significantly higher in the ulcerative colitis patients compared to controls.

**Table 1:** Baseline comparison of ulcerative colitis patients and controls * significant at a p-value of <0.05 ESR - erythrocyte sedimentation rate, CRP - C-reactive protein

Parameters	Ulcerative colitis cases (n=76)	Controls (n=76)	p-value
Age	39.68±10.07	38.23±8.34	0.834
Vitamin D (ng/ml)*	23.40±6.23	39.89±6.97	0.03
BMI	19.12±3.03	20.34±3.45	0.726
ESR*	23.29±7.17	13.23±9.23	0.042
CRP	23.40±6.23	9.23±6.11	0.027

Table 2 compares the characteristic of male and female cases. Age at time of diagnosis and BMI were significantly higher in females compared to males. There was no significant difference in disease activity and quality of life score between males and females.

**Table 2:** Gender wise distribution of baseline character of patients enrolled in the study * significant at a p-value of ≤0.05 # median and inter-quartile range given UCDAI - ulcerative colitis disease activity index, SIBDQ - short inflammatory bowel disease questionnaire, ESR - erythrocyte sedimentation rate, CRP - C-reactive protein

Parameters	Male (n=58)	Female (n=18)	Total patients	p-value
Age*	37.47±9.7	48±6.6	39.68±10.07	0.000
BMI*	18.24 ±2.52	22.39±2.57	19.12±3.03	0.000
UCDAI#	8.00 (6-10)	8.00 (5-9)	8.00 (5-11)	0.916
SIBDQ#	43.5 (20-51)	44 (29-52)	44 (20-52)	0.319
ESR	23.70±7.45	21.75±5.94	23.29±7.17	0.337
CRP	28.66±18.16	25.00±15.00	23.89±17.51	0.461
Vitamin D (ng/dl)	23.33±6.39	23.66±5.79	23.40±6.23	0.852

Rectal bleeding was the most common manifestation (93.3%), followed by tenesmus (73.6%) and abdomen pain (54%). 22.4% of the patients presented with pancolitis (Table 3).

**Table 3:** Key presenting symptoms and extent of disease of Ulcerative colitis in newly diagnosed patients

Symptoms	Number (%)
Presence of bleeding	71(93.3%)
Presence of tenesmus	56(73.6%)
Presence of pain	41(54%)
Extent of disease	
Pancolitis	17(22.4%)
Left-sided colitis	59(77.6%)

Table 4 and Table 5 show the association of vitamin D status with disease activity index and quality of life scores, and various inflammatory parameters. A total of 60 (78.9%) newly diagnosed ulcerative colitis patients had low vitamin D levels. Nearly one-quarter of the patients (23.3%) with low vitamin D had disease activity scores of more than nine compared to only 10 percent in normal vitamin D patients (p=0.030).

**Table 4:** Association of disease activity index and quality of life with vitamin D levels UCDAI - ulcerative colitis disease activity index, SIBDQ - short inflammatory bowel disease questionnaire

	Normal vitamin D (>30ng/dl)	Low vitamin D (<30 ng/dl)	p-value
UCDAI			
>9	2 (10%)	14 (23.3%)	0.030
≤9	14 (90%)	46 (76.7%)	
Quality of Life score (Assessed by SIBDQ)			
≥50	10 (70%)	7 (11.7%)	0.000
<50	6 (30%)	53 (88.3%)	

**Table 5:** Comparative analysis of ulcerative colitis patient with normal or low vitamin D status UCDAI - ulcerative colitis disease activity index, SIBDQ - short inflammatory bowel disease questionnaire, ESR - erythrocyte sedimentation rate, CRP - C-reactive protein * significant at a p-value of ≤ 0.05 # median and interquartile range given

Parameters	Normal vitamin D group (16)	Low vitamin D group (60)	p-value
Age	36.75±6.66	40.47±10.72	0.093
BMI	19.88±2.57	18.91±3.13	0.258
UCDAI*#	6.00(5-7)	8.50(6-10)	0.001
SIBDQ*#	50(47-51)	41.5(22-44)	0.000
ESR	22.63±8.69	23.47±6.7	0.723
CRP*	6.86±2.21	33.50±15.39	0.000

The median UCDAI score was significantly higher (p-0.030) in those with vitamin D deficiency, and quality of life was also worse in the vitamin D deficient group (p<0.000). Among inflammatory markers, C-reactive protein (CRP) was significantly higher in the group with low vitamin D group, but no such difference was observed in erythrocyte sedimentation rate (ESR).

Table 6 shows multiple linear regression analyses depicting the independent predictors of disease activity index and HRQOL. The disease activity index decreased with an increase in vitamin D levels. We had included age, BMI, ESR, CRP as other independent predictors in our model. Quality of life score also increased with an increase in vitamin D levels. Age, BMI, and sex were entered into the regression model as other independent predictors apart from vitamin D levels.

**Table 6:** Multiple linear regression analysis to show independent predictors of disease activity index and quality of life UCDAI - ulcerative colitis disease activity index, ESR - erythrocyte sedimentation rate, CRP - C-reactive protein

Parameters	Unstandardized coefficient (β)	Standardized coefficient	Confidence intervals	p-value
UCDAI				
Age	0.000	-0.001	-0.014 to 0.014	0.983
BMI	0.069	0.129	0.018 to 0.120	0.009
ESR	-0.005	0.023	-0.023 to 0.013	0.558
CRP	0.064	0.693	0.037 to0.091	0.000
Vitamin D	-0.083	-0.318	-0.156 to -0.010	0.026
Quality of Life				
Age	-0.092	-0.096	-0.18 to -0.001	0.047
BMI	0.548	0.172	0.203 to 0.893	0.002
Sex	-1.712	-0.073	-4.205 to 0.780	0.175
Vitamin D	1.277	0.824	1.130 to 1.425	0.000

## Discussion

The literature has shown considerable heterogeneity regarding a standard definition for vitamin D deficiency. It is generally accepted to determine vitamin D status by measuring serum 25 hydroxyvitamin D (25(OH)D). The currently accepted definition of vitamin D sufficiency was as measured by 25[OH]D is 30 ng/dL. Anyone below this level was considered to have low vitamin D levels [10]. Recent estimates show that although the prevalence of inflammatory bowel disease may be low in India, the absolute numbers may be very high due to our huge population [13,14]. The majority of the patients in our study were males, with the male to female ratio being more than 3. Most studies from India have consistently shown a slight preponderance of males compared to females [13,15-16]. The IBD survey in India reported a male to female ratio of 1.4 and 1.3 for UC and Crohn's disease (CD), respectively [13]. In another study by Sood et al., conducted in Punjab, the male to female ratio was 7.8:1 [15]. In a retrospective study by Ulitsky et al. conducted at the University of Wisconsin among IBD patients, females were higher than males [17]. A pooled analysis of the study in western countries has also not shown any major difference in sex distribution [18]. The preponderance of males in our study may be attributed to sociocultural factors prevalent in India, where females from low socioeconomic status tend to neglect the symptoms and report late to the hospital. A more detailed community-based survey or analysis is required to know the true epidemiological distribution of UC in the Indian population. The mean age of diagnosis in this study was 39.68 years, and it was significantly higher in males compared to females. The mean age was similar in an Indian task force study and a survey conducted in a northern state of India [13,15]. Studies from other parts of the world have shown mean age of diagnosis ranging from 30-40 years [16,19-20]. The median age of diagnosis of ulcerative colitis in a cohort of ulcerative colitis across 8 Asian countries was 37 years [16]. Inflammatory markers like CRP and ESR were elevated in cases at the time of the diagnosis in this study. There was no significant gender difference in the inflammatory biomarkers. In the present study, mean vitamin D levels in ulcerative colitis patients were 23.40 ng/dl at the time of diagnosis, and no significant difference was observed among the male and female gender.

Pancolitis was seen in 17 (22.4%) patients, and 59 (77.6%) had left side colitis. In the Indian Task Force data, almost 43% had pancolitis, and left-sided colitis was seen in 39% [13]. The fact that we had enrolled only newly diagnosed cases in our study could account for the lower number of pancolitis patients seen in our study when compared to the Indian task force study [13]. Studies from Europe have often shown left-sided colitis to be more common [19,20]. In a study conducted in 9 countries of the Asia-pacific region, proctitis has been seen in more than one-third of patients [21]. It has to be emphasized the extent of disease is not constant and may change with time.

Vitamin D and disease activity index and quality of life

In the present study, we have found that UC patients with low vitamin D levels were found to have significantly higher median UCDAI scores (8.5) compared with patients with normal vitamin D levels (6.). Among UC patients with low vitamin D, approximately one-fourth (23.3%) had UCDAI score >9, indicating severe disease. We did a regression analysis to find out the independent predictors of disease activity scores (Table 5). We entered age, BMI, CRP, ESR, and serum vitamin D in the regression model. UCDAI decreased with an increase in vitamin D levels, and this was significant.

In a study done by Zammit et al. among the European population, the disease activity index increased with decreased levels of vitamin D [22]. The study had evaluated disease activity using a simple clinical colitis activity index. Blanck et al. have also found a similar relationship between disease activity and vitamin D level [23]. A similar type of study done by Datt et al. among the Indian population had also shown a negative co-relation of disease activity with Vitamin D levels [24]. Median vitamin D levels were lower in the patient with a disease activity index of more than 6. In contrast to our study, higher vitamin D levels were found in patients with active ulcerative colitis compared to inactive cases in a study done by Hasan et al. [25]. The sample consisted of only 24 patients, and thus this small sample might explain the variability.

This study showed that the quality of life as assessed by a short inflammatory bowel disease questionnaire was worse in those with low vitamin D levels. All the patients with low vitamin D levels had a quality of life score of less than 50. In a study conducted by Halvaty et al., which evaluated the quality of life after 3 months of vitamin D supplementation, it was observed that quality of life was better in patients who had normal vitamin D compared to those who were deficient [26]. Another study conducted among the Portuguese population had also shown that IBD patients with SIBDQ scores <50 had significantly lower mean vitamin D levels compared with patients who had SIBDQ scores ≥50 [27]. A randomized control trial has shown to improve the quality of life of ulcerative colitis patients who were put on vitamin D supplementation [28]. This study was only a cross-sectional analysis, and no post-interventional analysis has been done in it.

We also did a multiple regression analysis to find out important independent predictors of disease activity index and quality of life. Increasing levels of vitamin D decreased the disease activity index and improved the quality of life. There is a paucity of studies done among the Indian population which have evaluated the independent role of vitamin D on disease activity index and quality of life in ulcerative colitis patients.

 

This study did have some limitations. This was a time-bound study, and we included only those ulcerative colitis patients who reported to the department of gastroenterology during a time frame of one year. No sample size calculation was done. Our controls consisted of patients who had reported to the outpatient department and were evaluated for ulcerative colitis but did not have it. But considering the fact, many patients reporting to the gastroenterology department may have other diseases which might have influenced their vitamin D levels, like low dietary intake. Quality of life is also likely to get influenced by multiple other factors. This was a cross-sectional study, and we can just comment on the association of vitamin D levels with ulcerative colitis disease life and not whether the levels of vitamin D were a cause or consequence of the disease.

## Conclusions

The role of vitamin D as an immunomodulator is well established. Our study showed that deficiency of vitamin D levels was associated with increased diseased activity and poor quality of life. The study also showed that patients with ulcerative colitis had a significantly lower level of vitamin D compared to those without the disease. Prospective studies are required to see whether vitamin D levels are influenced by the progression and extension of disease. Randomized control trials can establish the therapeutic benefits of vitamin D in the remission of ulcerative colitis.
